# Rectification: Best abstracts of the NVMO conference

**DOI:** 10.1007/s40037-013-0053-4

**Published:** 2013-04-10

**Authors:** Nele Michels

**Affiliations:** Skills Lab, Faculteit Geneeskunde en Gezondheidswetenschappen, Universiteitsplein, 1 – R3.14, 2610 Antwerpen, Wilrijk Belgium

In the previous edition of *Perspectives on Medical Education* the award winning and nominated abstracts of the yearly conference of the Netherlands Association of Medical Education were presented. We regret that one of the nominated abstracts was left out by mistake. The missing abstract by Nele Michels is presented below.

Bohn Stafleu van Loghum

**Nominated Abstract**

**A systematic review of validity issues related to portfolio use: hit the target and don`t miss the point.**

*Michels NRM* ^*1*^, *Remmen R* ^*1*^, *Denekens J* ^*1*^, *van Rossum H* ^*2*^, *de Winter BY* ^*1*^

^*1*^ *University of Antwerp, Antwerp, Belgium,* ^*2*^ *VU University Medical Center, Amsterdam, the Netherlands*

**Background:** Portfolios are used as instruments to support learning, competence achievement, and reflection [1]. When portfolios are used for assessment purposes, validity of portfolio data is paramount. This systematic review was performed to investigate in which way validity of portfolio data in medical education are studied and reported in scientific literature. The validity framework of Messick was used to investigate the various aspects of validity [2]. We aimed to formulate guidelines for future validity research of portfolios in the field of medical education.

**Method:** Medline, Embase and ERIC were systematically searched from 1999 to 2010, with no restriction on language and geography. Search terms were related to “portfolio”, “valid(ity)” and “medical education”. Titles and abstracts (2 researchers) and full articles (5 researchers) were double screened against inclusion and exclusion criteria. To be included the manuscripts needed to investigate the validity of educational portfolios [1] in settings related to medical, nursing or dental education. A BEME data coding form was used to collect data concerning context- and portfolio-related characteristics, study design and -quality, and the results of the validity studies. Next, the studies were classified according to the validity framework used by Messick [2] based on the methodology used. As such, different sources to prove evidence of validity could be disentangled.

**Results:** Eleven manuscripts could be included. In these studies, the description of the portfolios used and their contexts were clear (55 %) to moderately clear (45 %). The methodology used to evaluate validity was, on the contrary, in most cases of minor quality, regarding as well the study design and -execution as the used statistics. Portfolio scores (54 %) and perceptions of students and teachers on the portfolio (40 %) were mainly used to report validity, next to the description of the context and the portfolio per se (7 %). Most of the included studies (81 %) did not refer to an existing validity framework and approached validity as a vague concept.

**Discussion and conclusion:** Due to the moderate context descriptions, the use of the concept validity as a container term, and the weak research designs, the point in validity research on portfolio scores is often missed. For future work we recommend to include a detailed and clear description of the context in which the portfolio is embedded, and we suggest sound scientific research of the different validity evidence sources based on existing validity frameworks, e.g. the framework as described by Messick [2].

**References**

1. Buckley S, Coleman J, Davison I, et al. The educational effects of portfolios on undergraduate student learning: A Best Evidence Medical Education (BEME) systematic review. BEME Guide No. 11. Medical Teacher 2009; 31:282–98.

2. Downing SM. Validity: on the meaningful interpretation of assessment data. Medical Education 2003;37:830–7.

